# A new species of spider fly in the genus *Sabroskya* Schlinger from Malawi, with a key to Acrocerinae world genera (Diptera, Acroceridae)

**DOI:** 10.3897/zookeys.171.2137

**Published:** 2012-02-24

**Authors:** Shaun L. Winterton, Jéssica P. Gillung

**Affiliations:** 1 California State Collection of Arthropods, California Department of Food & Agriculture, Sacramento, California, USA; 2 Universidade de São Paulo, Departamento de Zoologia, Instituto de Biociências, São Paulo, SP, Brazil

**Keywords:** Acroceridae, spider parasitoid

## Abstract

In this paper we diagnose the genus *Sabroskya* Schlinger, 1960 and describe *Sabroskya schlingeri* **sp. n.** from Malawi. We also provide dichotomous keys to species of *Sabroskya* and to world genera of the subfamily Acrocerinae, both extant and extinct.

## Introduction

Spider flies (Diptera: Acroceridae) are a geographically cosmopolitan group although most species are relatively rarely collected. Adults have a distinctive morphology and a wide diversity of form, but typically with a small head, greatly enlarged lower calypter and swollen abdomen. Larvae are parasitoids of spiders, with a hypermetamorphic life cycle consisting of four instars (Schlinger 1981, 1987).

Acroceridae comprise approximately 520 species in 53 genera (Pape and Thompson 2011; Gillung and Winterton 2011) occupying most biogeographic regions. The family is presently classified in three extant subfamilies based on adult morphology and host specificity with Panopinae suggested as the most primitive and Acrocerinae the most derived, with Philopotinae supposedly occupying an intermediate position (Schlinger 1987; Schlinger 2009). Recent phylogenetic analyses using DNA sequence data suggest an opposite sequence of cladogenesis and that Acrocerinae are polyphyletic (Winterton et al. 2007).

Acrocerinae comprise 17 extant and 5 extinct described genera, found in all major biogeographical regions. The subfamily is distinguished from Philopotinae and Panopinae by the following characteristics: antennae styliform, postpronotal lobes widely separated, never medially contiguous, humeral crossvein rarely well developed, and tibial apical spines absent (rarely present) (Winterton in press). In phylogenetic analyses of DNA sequences for six sampled genera by Winterton et al. (2007), *Acrocera* Meigen, 1803 and *Sphaerops* Philippi, 1865 were recovered as a sister clade to the rest of Acroceridae. The remaining acrocerine genera sampled (i.e. *Pterodontia* Gray 1832, *Ogcodes* Latreille, 1797, *Turbopsebius* Schlinger, 1972, *Psilodera* Gray, 1832, *Holops* Philippi, 1865) were recovered in a monophyletic clade sister to Panopinae. *Acrocera* displays very different adult and larval morphology from all other acrocerids, supporting this conclusion. Yet, the placement of *Sphaerops* as sister to *Acrocera* is problematic as the adult morphology is more similar to *Villalus* Cole, 1966 than to *Acrocera* and should be re-examined using both morphology and DNA sequence data.

Six genera of Acrocerinae are known from the Afrotropical Region, including the nearly cosmopolitan genera *Acrocera*, *Ogcodes* and *Pterodontia*, as well as the endemic genera *Psilodera*, *Meruia* Sabrosky, 1950 and *Sabroskya* Schlinger, 1960. *Sabroskya* includes two previously described species from South Africa (*Sabroskya ogcodoides* Schlinger, 1960 and *Sabroskya palpalis* Barrclough, 1984) (Schlinger 1960a; Barraclough 1984) and can be readily identified from all other acrocerine genera by the presence of a cervical collar, antennae located adjacent to mouthparts, wing vein R_4+5 _straight, cell m_3_ absent and discal and basal r_4+5 _cells separate and closed. Herein we describe a new species of *Sabroskya* from Malawi and present a key to species. A key to living and fossil genera of Acrocerinae of the world is also presented.

## Materials and methods

Terminology follows McAlpine (1981) and Schlinger (1981) as modified by Winterton (in press). The type specimen is deposited in the collection of the Tel Aviv University (TAU). Specimen images were taken at different focal points using a digital camera and subsequently combined into a serial montage image using Helicon Focus software. High-resolution digital images were deposited into Morphbank:: Biological Imaging with embedded URL links within the document between descriptions and Morphbank images. All new nomenclatural acts and literature are registered in Zoobank (Pyle and Michel 2008).

## Taxonomy

### Key to Acrocerinae genera of the World:

The extinct genus *Juracyrtus* Nartshuk, 1996 is not included as it is represented by a compression fossil and lacks sufficient detail to be thoroughly differentiated from other genera. Two recently described genera, *Schlingeromyia* Grimaldi & Hauser, 2011 and *Burmacyrtus* Grimaldi & Hauser, 2011, from Cretaceous aged amber (Grimaldi et al. 2011) are included here in Acrocerinae based on the presence of stylate antennae, non-arched body shape and widely separated postpronotal lobes. The placement of *Burmacyrtus* in Acroceridae is problematic and should be reassessed as this genus lacks characters typical of acrocerids, including a mediolobus and wing crossvein 2r-m, and has a relatively small calypter. Based on these characters, placement in Heterodactyla should be considered rather than in Acroceridae, although a stem-group position for the genus as suggested by Grimaldi et al. (2011) may also be reasonable.

**Table:** 

1	Cell m_3_ present and well formed (Fig. 1A)	2
–	Cell m_3_ clearly absent (Figs 1B, 2–3), *or*, fusion of m_3_ with discal cell indicated by presence of spur veins (rare)	10
2	Antennae not adjacent to the ocellar tubercle; located on middle of frons, separated from ocellar tubercle by distance much greater than length of ocellar tubercle (Figs 3C, 5)	3
–	Antennae adjacent to the ocellar tubercle	5
3	Wing vein R_4+5 _forking from R_2+3 _in distal half of cell r_4+5_; cells bm and br fused into a single cell; cell r_4+5 _relatively broad; eye emarginate (Burmese Amber)	*Schlingeromyia* Grimaldi & Hauser, 2012
–	Wing vein R_4+5 _forking from R_2+3 _before or at base of cell r_4+5_; cells bm and br separate; cell r_4+5 _relatively narrow along entire length; eye not emarginate	4
4	Eyes apilose; radial veins curved anteriorly, joining to anterior margin of wing (Southern Africa)	*Psilodera* Gray, 1832
–	Eyes pilose; radial veins relatively straight, joining wing apex (Chile)	*Holops* Philippi, 1865
5	Eyes very sparsely pilose, few microscopic setae present (India)	*Subcyrtus* Brunetti, 1926
–	Eyes densely pilose	6
6	Mouthparts longer than head; palpi present; proboscis not pilose	7
–	Mouthparts shorter than head; palpi apparently not present; proboscis pilose (Fig. 3C)	8
7	Antennae separated form ocellar tubercle by small depression (Europe) (Fig. 1A [wing])	*Cyrtus* Latreille, 1797
–	Antennae not separated from ocellar tubercle by depression (China)	*Paracyrtus* Schlinger, 1972
8	Mouthparts very short, barely protruding from oral cavity (Palaeartic)	*Asopsebius* Nartshuk, 1982
–	Mouthparts longer, protruding from oral cavity, but not longer than head	9
9	Labellum present; abdominal spiracles II - IV placed in intersegmental membranes (Taiwan)	*Hadrogaster* Schlinger, 1972
–	Labellum absent; abdominal spiracles II - IV placed in corresponding sternites (Taiwan and Japan)	*Nipponcyrtus* Schlinger, 1972
10	Antennae located on upper half of head, usually proximal to ocellar tubercle	11
–	Antennae located on lower half of head, adjacent to oral cavity	17
11	Vein R_4+5 _represented as a single unforked vein	12
–	Veins R_4 _and R_5_ forked and petiolate basally (R_4_ rarely incomplete basally)	14
12	Eyes minutely pilose, setae barely evident; petiolate to wing margin; flagellum with minute terminal seta; male genitalic capsule enlarged and bulbous (Chile) (Fig. 1B)	*Sphaerops* Philippi, 1865
–	Eyes clearly pilose; flagellum with relatively large terminal seta; male genitalic capsule not enlarged or bulbous	13
13	Microtrichia on the wing membrane absent; A_1_ joined to wing margin separate from CuA_2_ (Baltic Amber)	*Villalites* Hennig, 1966
–	Microtrichia on the wing membrane present; A_1_ and CuA_2_ approximated distally but incomplete, not joined to wing margin (Chile)	*Villalus* Cole, 1918
14	Wing with single medial vein (M_3_?); cell bm only well defined, other cells reduced or merged to form single cell open basally; alula well developed (most biogeographic regions)	*Acrocera* Meigen, 1803
–	Wing with three medial veins originating from discal cell; wing with three or four wing cells well defined; alula present or absent	15
15	Mediolobus absent; crossvein 2r-m absent so that only three closed wing cells present; antennal style longer than rest of flagellum (Burmese Amber)	*Burmacyrtus* Grimaldi & Hauser, 2011
–	Mediolobus present and similar shaped to pulvilli; crossvein 2r-m present so that four closed wing cells are present; antennal style shorter than rest of flagellum	16
16	Anterior ocellus reduced but present; costa circumambient; male wing with anterior costal process (Nearctic)	*Turbopsebius* Schlinger, 1972
–	Anterior ocellus absent; costa ending in radial field near wing apex; male wing without anterior process (Palaearctic)	*Opsebius* Costa, 1855
17	Wing with remnants of cell m_3_ indicated by presence of spur veins in cell d+m_3_ (Hennig 1968: figs 5, 8) (Baltic Amber)	*Glaesoncodes* Hennig, 1968
–	Wing cell m_3_ not indicated by spur veins	18
18	Wing cells d and basal r_4+5 _separate; antepronotum produced anteriorly as collar-like process behind head (Schlinger 1960a: fig. 13)	19
–	Wing cells d and basal r_4+5 _(and m_3_) fused to form large single cell (Fig. 2B), or cells absent (Fig. 2A); antepronotum not forming collar-like process behind head	20
19	Thorax greatly enlarged dorsally; wing veins R_2+3 _and R_4+5 _curved anteriorly then reflexed towards wing apex; vein M_2_ reaching wing margin; alula absent (Kenya) (Sabrosky 1950: fig. 2a)	*Meruia* Sabrosky, 1950
–	Thorax rounded but not greatly enlarged; radial veins straight; vein M_2_ not reaching wing margin; alula present (southern Africa) (Figs 3–10)	*Sabroskya* Schlinger, 1960
20	Tibial spines present apically; mouthparts present (Cosmopolitan) (Fig. 2B)	*Pterodontia* Gray, 1832
–	Tibial spines absent; mouthparts absent, oral cavity closed (Cosmopolitan) (Fig. 2A)	*Ogcodes* Latreille, 1797

**Figure 1.**
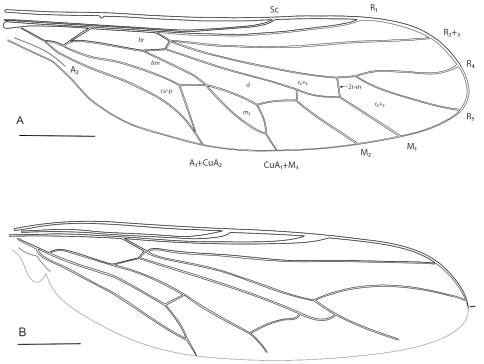
Acroceridae wings. Acrocerinae: **A** *Cyrtus gibbus* (Fabricius, 1794) **B** *Sphaerops* *appendiculata* Philippi, 1865. Scale line = 0.2 mm.

**Figure 2.**
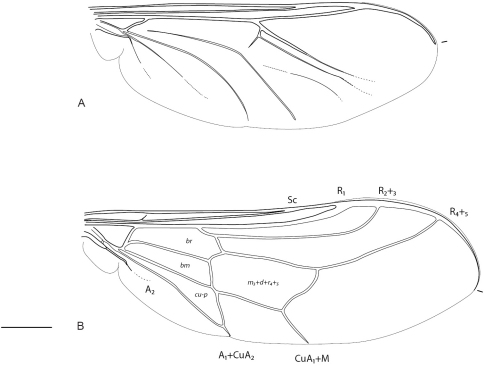
Acroceridae wings. Acrocerinae: **A** *Ogcodes basalis* Walker, 1852 **B** *Pterodontia davisi* Paramonov, 1957 (female). Scale line = 0.2 mm.

#### Sabroskya

Schlinger

http://species-id.net/wiki/Sabroskya

##### Type species.

*Sabroskya ogcodoides* Schlinger 1960: 479 by original designation.

##### Diagnosis.

Body length: 6.0–7.0 mm. Body shape not arched. Head width slightly narrower than thorax; sub-spherical; postocular ridge and occiput rounded; three ocelli; posterior margin of eye rounded; eye pilose (dense); eyes contiguous above antennal base; antennae located adjacent to mouthparts; palpus present or absent; proboscis length less than head length, with sparse setal pile; flagellum stylate, apex with relatively large terminal seta; postpronotal lobes not enlarged or contiguous medially; antenotum expanded, collar-like behind head; subscutellum relatively enlarged; tibial spines absent; pulvilli present; wing hyaline or slightly smoky infuscate, markings absent; costa ending near wing apex; costal margin straight; humeral crossvein absent; R_1_ very slightly inflated at pterostigma; R_2+3 _present or absent; veins R_4_ and R_5_ present as single vein R_4+5_; radial veins straight, complete to wing margin; crossvein 2r-m present between M_1 _and R_4+5_, bisecting cell r_4+5_, cell formed by 2r-m narrow elongate; medial vein compliment: M_1_, M_2_ and M_3_ present (M_3_ fused with CuA_1_), medial veins may or may not reach wing margin; discal cell closed completely; cell m_3_ absent; CuA_2_ fused to A_1_ before wing margin, petiolate; wing microtrichia absent; anal lobe well developed; alula well developed; abdominal tergites smooth, rounded; abdomen rounded, inflated, slightly wider than thorax.

##### Comments.

*Sabroskya* is a highly specialized Acrocerinae spider fly genus morphologically similar to *Meruia*, *Ogcodes*, *Glaesoncodes* and *Pterodontia*. These five genera all have stylate antennae located on the lower side of the head adjacent to the often reduced or absent mouthparts. Other acrocerine genera related to this clade include *Turbopsebius*, *Opsebius*, *Villalus*, *Acrocera* and *Sphaerops*, all of which have a wing venation lacking cell m_3_. The Baltic amber genus *Glaesoncodes* is unique among this acrocerine clade as the wing retains remnants of cell m_3_, with spur veins present in cell d+m_3_ (Hennig 1968); similar remnants of m_3_ can also be found in more distantly related *Turbopsebius*. This provides important insights into the evolution of acrocerid wing venation, suggesting rampant reduction in number of cells and veins through loss or fusion, and can be found in derived clades in all three extant subfamilies (Winterton et al. 2007; Gillung and Winterton 2011).

**Figure 3.**
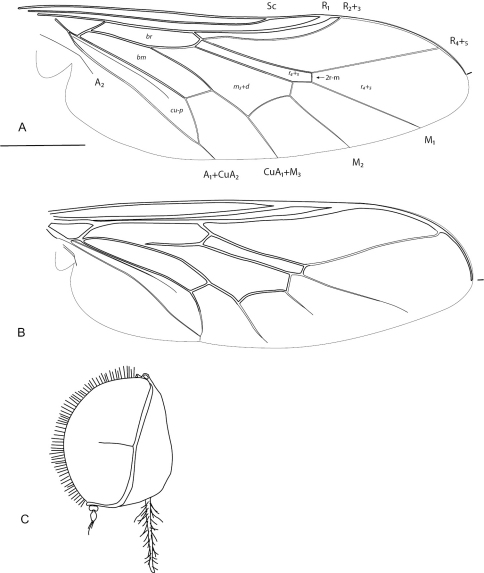
Acrocerinae: A, *Sabroskya ogcodoides* Schlinger, 1960a; B, *Sabroskya schlingeri* sp. n.; C, *Sabroskya schlingeri* sp. n., male head, lateral view. Scale line = 0.2 mm.

In *Pterodontia*, *Sabroskya* and *Ogcodes* the costal margin has a membranous rim or flange between R_1_ and wing apex (Figs 2–3). This character still needs to be confirmed in *Meruia*, but appears to be likely a synapomorphy for the group. The putative sister genus to *Sabroskya* is *Meruia*, and both have similar wing venation comprising well defined and complete discal and basal r_4+5 _wing cells. These cells are absent in *Ogcodes* and are fused to form a single cell in *Pterodontia*. *Sabroskya* can be immediately identified from other acrocerine genera by the presence of a cervical collar, antennae located adjacent to mouthparts, R_4+5 _straight, cell m_3_ absent and discal and basal r_4+5 _cells separate and closed.

Schlinger (1960a) described the antennal flagellum of *Sabroskya* as stylate without a terminal seta, and with a large subterminal seta on the lateral surface of the flagellum. Detailed examination of the topotype series of *Sabroskya ogcodoides* (Schlinger 1960b) shows a similar condition as found in both *Sabroskya schlingeri* sp. n. and *Sabroskya palpalis*, with the flagellum actually having large terminal setae present (Fig. 3C) (see also Grimaldi (1995: fig. 5)). Only in *Sabroskya palpalis* are palpi present while in *Sabroskya ogcodoides* and *Sabroskya schlingeri* sp. n., the palpi are absent.

##### Included species.

*Sabroskya ogcodoides* Schlinger, 1960; *Sabroskya palpalis* Barraclough, 1984; *Sabroskya schlingeri* sp. n.

##### Key to species of *Sabroskya*..

(Females are unknown for *Sabroskya palpalis* and *Sabroskya schlingeri* sp. n.)

**Table:** 

1	Flagellum with subterminal setae absent; palpi present; posterior surface of hind coxae apilose; paler areas of male abdominal tergites not connected medially (South Africa)	*Sabroskya palpalis* Barraclough, 1984
–	Flagellum with subterminal setae present (Fig. 3C); palpi absent; posterior surface of hind coxae pilose; paler areas of male abdominal tergites connected medially	2
2	Male wing venation brown; vein R_2+3 _absent (Fig. 3B); wing smoky infuscate anteriorly; thoracic, abdominal and lower calypter pile dark (Malawi) (Figs 3–6)	*Sabroskya schlingeri* sp. n*.*
–	Male wing venation white, brown in female; vein R_2+3 _present (Fig. 3A); wing hyaline; thoracic, abdominal and lower calypter pile white (South Africa) (Figs 7–10)	*Sabroskya ogcodoides* Schlinger, 196*0*

#### Sabroskya schlingeri sp. n.

urn:lsid:zoobank.org:act:1C52AA02-CD70-4B6E-B2E9-F236A6C1DEB4

http://species-id.net/wiki/Sabroskya_schlingeri

Figures 3B, 3C 4 [Fig F5] -6

##### Type material.

**Holotype** male, MALAWI: Northern Province: North Viphya Mts, 1500 m, Rt. M1, 21–22.ix.1998, 10 km S Chikangawa [-11.929, 33.747], F. Kaplan, A. Freidberg (TAU).

##### Diagnosis.

Wing venation black; vein R_2+3 _absent; wing hyaline, smoky infuscate anteriorly; flagellum with subterminal seta present; lower calypter pile short, dark; thoracic and abdominal pile black; palpi absent; hind coxae with setae on posterior surface; paler areas of abdominal tergites connected medially.

##### Description.

Body length 5.0 mm (male). *Head*. Eye brown, densely pilose with setae approximately length of tarsal claw; posterior margin of eye not emarginate; ocellar tubercle glossy black and raised around ocelli; occiput glossy black, coriacious, pile black; postocular ridge, gena to parafacial with narrow grey pubescent ridge; palpus absent; margin of oral cavity apilose; proboscis shorter than head length (Fig. 3C); antenna brown; flagellum apex with relatively elongate terminal seta, subterminal seta(e) present laterally. *Thorax*. Scutum glossy black with bronze suffusion anteriorly, postalar callus yellowish; vestiture as dense brown-black pile, paler on postalar callus; scutellum glossy black with dense black pile; pleuron glossy black with brown to yellowish pile; coxae black with yellow pile; femora dark yellow with black suffusion basally, pile yellow; tibiae yellow with short yellow pile; tarsi yellow; lower calypter hyaline with darkish margin; pile on membrane and along rim yellow to brown; wing hyaline, slightly smoky infuscate anteriorly, venation dark; vein R_2+3 _absent (Fig. 3B); M_2_ very short. *Abdomen.* Elongate globose, slightly wider than thorax, tergites dark brown anteriorly, yellow laterally and meeting posteromedially; covered with brown-black setae, erect and tufted medially on each tergite. Male genitalia: not dissected, externally similar to *Sabroskya ogcodoides*.

##### Etymology.

The specific epithet is named in honor of Evert I. Schlinger, a foremost expert on world Acroceridae taxonomy and patron of dipterology. Evert Schlinger had previously identified that this specimen represented a new species of *Sabroskya*.

##### Comments.

*Sabroskya* *schlingeri* sp. n. is known only from a single male specimen from Malawi. A label on the pin of the holotype indicates that E. I. Schlinger had recognized that this species was a new taxon separate from the two previously described species. This is the most northern record for the genus, with both previously described species recorded from Eastern Cape and KwaZulu-Natal Provinces of South Africa. The lack of vein R_2+3_, dark vestiture and wing venation, and smoky infuscate wing readily differentiate this species from *Sabroskya palpalis* and *Sabroskya ogcodoides.*

**Figure 4.**
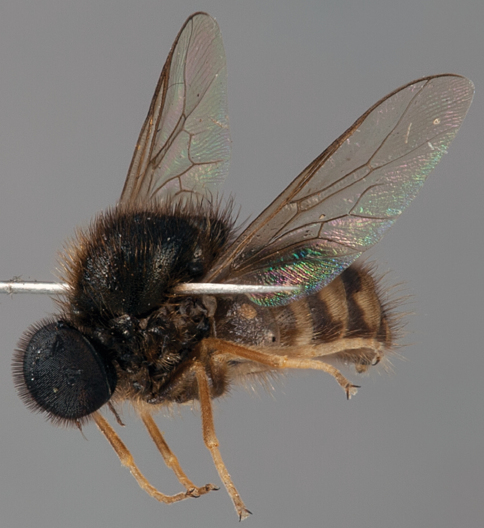
*Sabroskya schlingeri* sp. n., male, oblique view [Morphbank: 705550]. Body length = 5.0 mm.

**Figure 5. F5:**
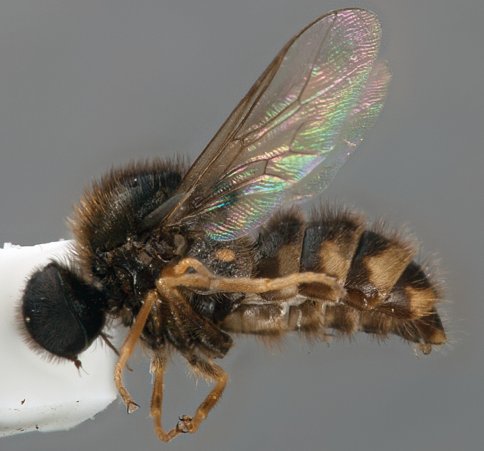
*Sabroskya schlingeri* sp. n., male, lateral view [Morphbank: 705551]. Body length = 5.0 mm.

**Figure 6.**
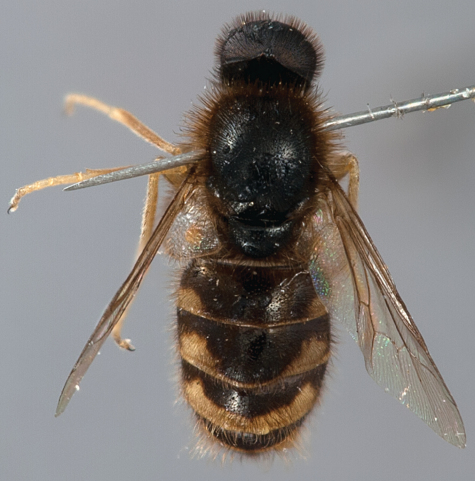
*Sabroskya schlingeri* sp. n., male, dorsal view [Morphbank: 705552]. Body length = 5.0 mm.

**Figure 7.**
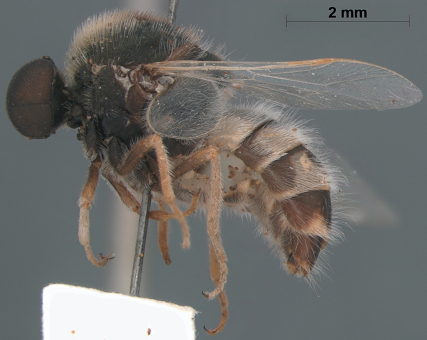
*Sabroskya ogcodoides* Schlinger, male, lateral view.

**Figure 8.**
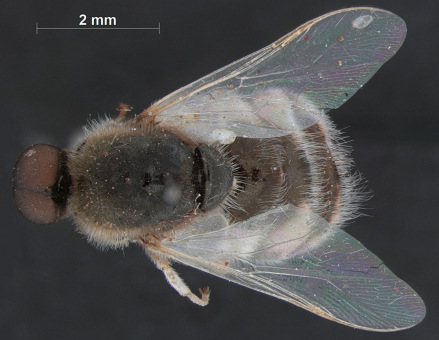
*Sabroskya ogcodoides* Schlinger, male, dorsal view.

**Figure 9.**
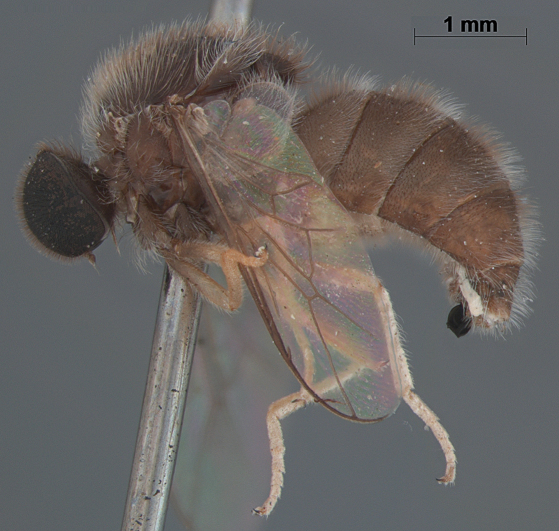
*Sabroskya ogcodoides* Schlinger, female, lateral view.

**Figure 10.**
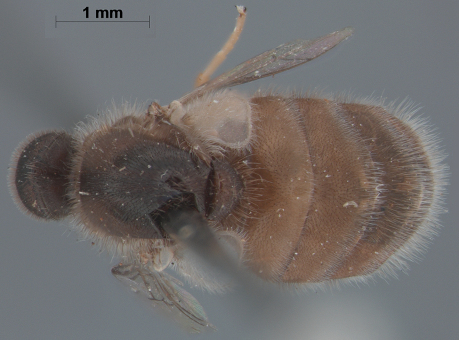
*Sabroskya ogcodoides* Schlinger, female, dorsal view.

## Supplementary Material

XML Treatment for Sabroskya

XML Treatment for Sabroskya schlingeri

## References

[B1] Barraclough DA (1984)Review of some Afrotropical Acroceridae, with descriptions of eight new species from South Africa (Diptera: Brachycera) . Journal of the Entomological Society of South Africa 47: 45–66 http://content.sabinet.co.za/u?/0012-8789,210

[B2] Gillung JP Winterton SL (2011) New genera of philopotine spider flies (Diptera: Acroceridae) with a key to living and fossil genera. Zookeys 127: 15-27 doi: 10.3897/zookeys.127.1824 21998545 10.3897/zookeys.127.1824 PMC3175128

[B3] Grimaldi DA (1995) A remarkable new species of *Ogcodes* (Diptera: Acroceridae) in Dominican amber. American Museum Novitiates 3127: 1-8

[B4] Grimaldi DA Arillo A Cumming JM Hauser M (2011) Brachyceran Diptera (Insecta) in Cretaceous ambers, Part IV, Significant New Orthorrhaphous taxa. Zookeys 148: 293-332 doi:10.3897/zookeys.148.1809 22287902 10.3897/zookeys.148.1809 PMC3264415

[B5] Hennig W (1968) Ein weiterer Vertreter der Familie Acroceridae im Baltischen Bernstein (Diptera: Brachycera). Stuttgarter Beiträge zur Naturkunde aus dem Staatlichen Museum 185: 1-6

[B6] McAlpine JF (1981) Morphology and terminology-Adults. In: McAlpine JF Peterson BV Shewell GE Teskey HJ Vockeroth JR Wood DM (Ed). Manual of Nearctic Diptera.Research Branch, Agriculture Canada Monograph 1: 9–63

[B7] Pape T Blagoderov V Mostovski MB (2011) Order Diptera Linnaeus, 1758. In: Zhang Z-Q, (Ed). Animal biodiversity: An outline of higher-level classification and survey of taxonomic richness.Zootaxa 3148: 222–229 http://www.mapress.com/zootaxa/list/2011/3148.html

[B8] Pyle RL Michel E (2008) Zoobank: Developing and nomenclatural tool for unifying 250 years of biological information. Zootaxa 1950: 39-50

[B9] Schlinger EI (1960a) A review of the South African Acroceridae (Diptera). Annals of the Natal Museum 14: 459–504 http://content.sabinet.co.za/u?/03040798,876

[B10] Schlinger EI (1960b) Additional notes on the South African acrocerid fauna, with descriptions of new species of *Acrocera* Meigen and *Psilodera* Gray (Diptera). Annals of the Natal Museum 15: 57–67 http://content.sabinet.co.za/u?/03040798,809

[B11] Schlinger EI (1981) Acroceridae. In: McAlpine JF Peterson BV Shewell GE Teskey HJ Vockeroth JR Wood DEM (Eds). Manual of Nearctic Diptera.vol. I. Research Branch, Agriculture Canada. Monograph 27: 575-584

[B12] Schlinger EI (1987) The biology of Acroceridae (Diptera): True endoparasitoids of spiders. In: Nentwig W (Ed) Ecophysiology of spiders. Springer Verlag, Berlin, xi + 319–327

[B13] Schlinger EI (2009) Acroceridae (spider flies, small-headed flies). In: Brown BV et al. (Eds) Manual of Central American Diptera: Volume 1 NRC Research Press, Ottawa, Ontario, Canada, 551–556

[B14] Winterton SL Wiegmann BM Schlinger EI (2007) Phylogeny and Bayesian divergence time estimations of small-headed flies (Diptera: Acroceridae) using multiple molecular markers. Molecular Phylogenetics and Evolution 43: 808-832 doi: 10.1016/j.ympev.2006.08.015 17196837 10.1016/j.ympev.2006.08.015

[B15] Winterton SL (in press) Review of Australasian spider flies (Diptera, Acroceridae) with a revision of *Panops* Lamarck. ZooKeys. 10.3897/zookeys.172.1889 PMC3307363 22448114

